# Radiation Exposure During Diagnostic and Therapeutic Angiography of Carotid-cavernous Fistula: A Retrospective Single Center Observational Study

**DOI:** 10.1007/s00062-021-01126-x

**Published:** 2021-12-21

**Authors:** Marcel Opitz, Georgios Alatzides, Sebastian Zensen, Denise Bos, Axel Wetter, Nika Guberina, Marvin Darkwah Oppong, Karsten H. Wrede, Tim Hagenacker, Yan Li, Isabel Wanke, Michael Forsting, Cornelius Deuschl

**Affiliations:** 1 Institute of Diagnostic and Interventional Radiology and Neuroradiology, Faculty of Medicine, University Hospital Essen, Hufelandstraße 55, 45147 Essen, Germany; 2 Department of Diagnostic and Interventional Radiology, Neuroradiology, Asklepios Klinikum Harburg, Hamburg, Germany; 3 Department of Radiation Therapy, University Hospital Essen, West German Cancer Center, Essen, Germany; 4 Department of Neurosurgery and Spine Surgery, University Hospital Essen, Essen, Germany; 5 Department of Neurology and Center for Translational Neuro- and Behavioral Science (C-TNBS), University Hospital Essen, Essen, Germany; 6 Department of Neuroradiology, Clinic Hirslanden, Zurich, Switzerland

**Keywords:** Radiation exposure, Carotid-cavernous fistula, Embolization, Interventional neuroradiology, Cerebral angiography

## Abstract

**Purpose:**

The aim of this study was to determine local diagnostic reference levels (DRLs) during endovascular diagnostics and therapy of carotid-cavernous fistulas (CCF).

**Methods:**

In a retrospective study design, DRLs, achievable dose (AD) and mean values were assessed for all patients with CCF undergoing diagnostic angiography (I) or embolization (II). All procedures were performed with the flat-panel angiography system Allura Xper (Philips Healthcare). Interventional procedures were differentiated according to the type of CCF and the type of procedure.

**Results:**

In total, 86 neurointerventional procedures of 48 patients with CCF were executed between February 2010 and July 2021. The following DRLs, AD and mean values could be determined: (I) DRL 215 Gy ∙ cm^2^, AD 169 Gy ∙ cm^2^, mean 165 Gy ∙ cm^2^; (II) DRL 350 Gy ∙ cm^2^, AD 226 Gy ∙ cm^2^, mean 266 Gy ∙ cm^2^. Dose levels of embolization were significantly higher compared to diagnostic angiography (*p* < 0.001). No significant dose difference was observed with respect to the type of fistula or the embolization method.

**Conclusion:**

This article reports on diagnostic and therapeutic DRLs in the management of CCF that could serve as a benchmark for the national radiation protection authorities. Differentiation by fistula type or embolization method does not seem to be useful.

## Introduction

Carotid-cavernous fistulas (CCFs) are abnormal communications between the carotid artery and its branches and the venous system of the cavernous sinus. Cerebral angiography is the gold standard for definitive diagnosis, classification and therapeutic planning [1]. Recent advances in endovascular technology have expanded the range of treatment options for CCFs and favorable long-term outcomes have been achieved [2–4]. As a result, the mainstay of treatment for CCFs consists of transarterial or transvenous embolization. Furthermore, the endovascular approach has evolved as the first-line treatment option in clinical emergencies and following failure of conservative therapy [5, 6]; however, other treatment options, such as open surgery or stereotactic radiosurgery are available as second-line or adjuvant therapeutic options [7, 8].

Neurointerventional procedures as diagnostic and therapeutic tools have increased significantly over the past decade. These minimally invasive fluoroscopy-guided procedures are an effective treatment option for various neurovascular diseases; however, due to the complexity of the pathologies being treated, some procedures may comprise high radiation exposure to patients as well as staff members [9–11], leading to an increased stochastic risk of developing radiation-induced cancer [12]. Several professional and regulatory organizations, such as the International Commission on Radiological Protection (ICRP), proclaim the necessity for diagnostic reference levels (DRLs) in regularly used neurointerventional procedures for quality control and benchmarking between institutions [13]. The goal is to raise awareness of dose and, in the long term, to optimize modification of equipment, techniques and imaging parameters [14–16].

Data on radiation exposure of diagnostic and therapeutic angiography in patients with CCF remain scarce. Hence, the aim of this study was to establish local DRLs at our department utilizing contemporary digital equipment.

## Methods

### Patient Cohort

This retrospective study was approved by the Ethics Commission of the Medical Faculty of the University of Duisburg-Essen (21-9944-BO) and was conducted in accordance with the principles of the Declaration of Helsinki. All procedures were performed after obtaining written informed consent. The internal angiographic database was searched with an in-house developed software for all consecutive diagnostic and therapeutic angiographies of CCF between February 2010 and July 2021. All CCFs were classified according to the Barrow classification (Table 1; [17]).

**Table 1:** Classification of the study population with carotid-cavernous fistula (CCF) according to the Barrow-classification

Type	Shunt pattern	*n* (%)
A	Direct connection between the intracavernous internal carotid artery and cavernous sinus	14 (29.2)
B	Dural shunt between intracavernous branches of the internal carotid and cavernous sinus	8 (16.7)
C	Dural shunts between meningeal branches of the external carotid artery and cavernous sinus	3 (6.2)
D	Combined type B + type C	23 (47.9)

### Procedure

All patients in this study cohort underwent diagnostic cerebral angiography (DCA) in-house or externally prior to embolization. The DCA was performed to confidently detect CCF, classify the fistula, and plan for possible embolization. The decision to perform endovascular intervention was based on a case by case evaluation in an interdisciplinary decision-making process between neurosurgeons and interventional neuroradiologists. At our department, the endovascular therapeutic approach for CCF depends on the type of fistula, the size of the arterial defect, and the surgeons’ preferences. In most cases, a transvenous approach was preferred and embolization was performed using coils and/or liquid embolic agent (Onyx®, Medtronic, Dublin, Ireland). In some cases, transarterial embolization was favored as an individual procedure or an additional balloon protection of the internal carotid artery was performed (Table 2). All angiographies were performed with the patient under general anesthesia.

**Table 2:** Demographic data and distribution of procedure type

Parameter	*n* (%)
*Number of patients*	48 (100)
*Male/female*	14 (29)/34 (71)
*Diagnostic angiographies*	26 (54)
*Number of embolizations*	60 (100)
With Onyx	7 (12)
With coils	23 (38)
With Onyx and coils	20 (33)
Additional balloon protection of internal carotid artery	9 (15)
Frustrated	10 (17)
Transarterial	12 (20)
Transvenous	48 (80)

### Biplanar Angiography System

All procedures were performed on the Allura Xper FD20/10 system (Philips Healthcare, Eindhoven, The Netherlands) by an experienced team of neuroradiologists. As we are a university hospital, young neuroradiologists were regularly involved in the interventions in addition to a neuroradiologist with many years of angiography experience. The X‑ray unit was equipped with an automatic exposure control system. The frame rate frequently used in the pulsed fluoroscopy mode was 1 pulse/s. The focus-to-skin distance varied from 60 to 70 cm. The Allura Xper system had one 20-inch detector with a maximum field of view (FOV) of 48 cm and one 10-inch detector with a maximum FOV of 25 cm. The minimum inherent filtration (at 75 kV) of the X‑ray tube/collimator was 2.5 mm Al. Besides a wedge filter of 1 mm brass (CuZn37 R‑019; 22 mm Al equivalent at 75 kV), an additional filter (0.9 mm Cu + 1.0 mm Al) was set, depending on the beam-limiting device. To test the performance and stability of the system over time, regular quality checks were performed during maintenance visits.

### Dose Calculation

Radiation exposure during diagnostic and therapeutic angiography was determined as dose area product (DAP). The ICRP recommends using the term DRL for both diagnostic and therapeutic interventional procedures [13]. To achieve dose optimization in the clinical routine, DRLs are a globally accepted element for dose monitoring, usually defined as DAP in interventional settings. The DRLs represent the 75th percentile of a dose distribution of a specific radiological procedure and may indicate whether the radiation dose lies within the normal range of a dose distribution at radiological departments [18, 19]. Achievable dose (AD) is another important parameter for dose optimization representing the median of a dose distribution [20].

### Statistical Analysis

The interventions were analyzed according to the type of procedure and the type of fistula. The mean, median and 75th percentile of the DAP, as well as the mean fluoroscopy time (FT) were calculated. A *p*-value below 0.05 was considered statistically significant. Statistical analysis was performed using SPSS (Statistical Package for Social Sciences) v. 27.0. (IBM, Armonk, NY, USA).

## Results

Between February 2010 and July 2021, 86 consecutive neurointerventional procedures were performed in 48 patients with CCF in our department. The median age of patients was 64 years (range 31–87 years). The gender distribution was clearly in favor of the female gender (34 females, 14 males). Out of 48 patients with CCF, 11 patients underwent more than 1 endovascular therapy. In 10 out of 60 procedures (16.7%), the embolization attempt had failed (Table 2).

**Fig. 1:**
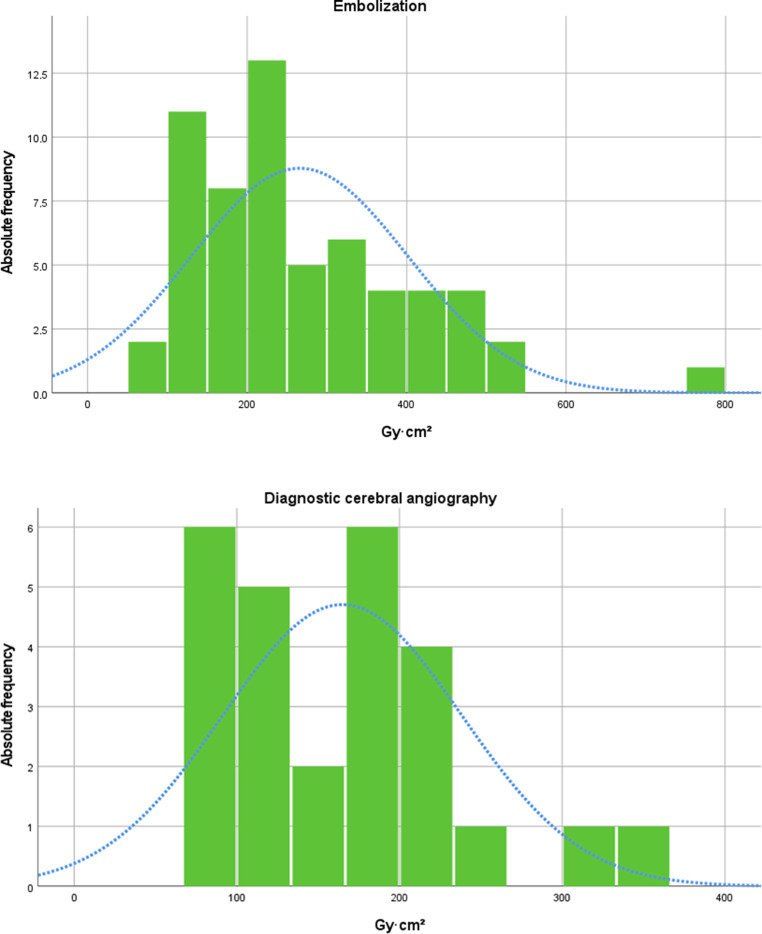
Histograms of distribution of dose area product (Gy ∙ cm^2^) for embolization (*upper*) and diagnostic cerebral angiography (*lower*) in patients with CCF; *blue* *curve* highlighting distribution graph

For all patients with CCF who underwent diagnostic angiography (I) or embolization (II), the following DRL, AD and mean values were obtained: (I) DRL 215 Gy ∙ cm^2^, AD 169 Gy ∙ cm^2^, mean 165 Gy ∙ cm^2^; (II) DRL 350 Gy ∙ cm^2^, AD 226 Gy ∙ cm^2^, mean 266 Gy ∙ cm^2^ (Table 3; Fig. 1).

**Table 3:** Distribution of total dose area product and fluoroscopic time of carotid-cavernous fistula as a function of procedure type

Type of procedure		Total DAP (Gy ∙ cm^2^)				FT (min)
	*n*	25th percentile	Median	75th percentile	Mean	Mean
Diagnostic	26	101.17	168.61	214.68	164.83	18.67
Embolization	60	155.72	225.68	349.51	265.84	61.90

*DAP* dose area product, *FT* fluoroscopic time, *n* number of studies

The results of one-way ANOVA confirmed a significant dose difference between DCA and embolization in patients with CCF (*p* < 0.001). The Kruskal-Wallis-test revealed no significant dose difference regarding the type of fistula according to the Barrow-Classification (*p* = 0.307) or the embolization method (*p* = 0.217). The results of the Mann-Whitney U test confirmed no significant dose difference between the procedures with both transarterial and transvenous access and the procedures with transarterial access only (*p* = 0.365).

Excluding the unsuccessful embolization attempts from all treatment sessions, no significant difference in DAP was observed (*p* = 0.232). Likewise, no significant difference in DAP was found regarding CCF embolization by excluding all patients who underwent more than one treatment session (*p* = 0.556). No significant FT difference was found between the type of fistula (*p* = 0.217) or the embolization method (*p* = 0.087). The mean FTs are listed in Table 3.

## Discussion

This retrospective single center study is the first to provide detailed dosimetry data for endovascular treatment of CCFs. In particular, the dedicated consideration of both Barrow grade and endovascular technique is unique. Therefore, it may be valuable to introduce novel DRLs in the field of interventional neuroradiology, as proposed by the European Directive 2013/59/Euratom [16].

In neuroradiology, a wide range of interventional procedures have been established over the last decade and are being used more frequently worldwide. The role of DRLs in interventional neuroradiology has increased in recent years and the guidelines for radiation protection have been updated to include interventional procedures that are regularly used in clinical routine [21–23].

Concerning interventional neuroradiology, the German Federal Office for Radiation Protection has so far only published DRLs for thrombus aspiration (DRL 180 Gy ∙ cm^2^) and aneurysm coiling (DRL 250 Gy ∙ cm^2^) [21]. Data on radiation exposure of other neuroradiological procedures remain scarce and to our knowledge no data have been published for CCF embolization. The local DRLs for CCF embolization in our study (350 Gy ∙ cm^2^) are substantially lower compared with published data on cranial AVM (DRL range 440–550 Gy ∙ cm^2^) [24, 25] and lateral dAVF embolization (DRL 414 Gy ∙ cm^2^) [26], although comparison is difficult because of differences in anatomic location and endovascular treatment techniques.

The use of DRLs in interventional radiology is challenging because of the high individual variability of procedures within the same type of procedure. In general, it is recommended to collect radiation data of more than 50 procedures within the same type of procedure to determine DRLs for a single center [27]. As previous studies have shown, radiation exposure for interventional procedures is much more affected by the complexity of the procedure than by the size and weight of the patient [28]. Therefore, DRL values for interventional procedures should ideally be set according to the type and level of complexity of the procedure. In general, therapeutic procedures have been reported to yield higher doses than diagnostic procedures [29]. Consequently, diagnostic and therapeutic DRLs should be defined separately.

The main limitation of our study is the retrospective and single center design. An experienced team of neuroradiologists performed all procedures; however, young neuroradiologists are also trained at our university hospital. Thus, in terms of radiation dose, our results could indicate higher doses than can be achieved. Moreover, the obtained dose levels could differ from those obtained at other sites and angiography devices. Consequently, investigation of radiation exposure in a larger population at different sites and devices in multicenter studies is the next necessary step for the establishment of national and European DRL values.

## Conclusion

The CCF embolization is a frequently used neurointerventional procedure and evolved as the leading curative therapeutic approach. Our results could serve as a benchmark for national radiation protection authorities to implement DRLs for CCF management. Differentiation by fistula type or embolization method does not seem to be useful.
